# MAP4K4 promotes ovarian cancer metastasis through diminishing ADAM10-dependent N-cadherin cleavage

**DOI:** 10.1038/s41388-023-02650-5

**Published:** 2023-03-15

**Authors:** Kelie Chen, Xiaoyu Yuan, Shengchao Wang, Fang Zheng, Zhiqin Fu, Zhangjin Shen, Xiaodong Cheng, Yuwei Wang, Song Tang, Heng Ni, Fang Wang, Guang Lu, Yihua Wu, Dajing Xia, Weiguo Lu

**Affiliations:** 1 Department of Gynecologic Oncology of Women’s Hospital, Zhejiang University School of Medicine, Hangzhou, Zhejiang Province China; 2 Department of Toxicology of School of Public Health, Zhejiang University School of Medicine, Hangzhou, Zhejiang Province China; 3 The Cancer Hospital of the University of Chinese Academy of Sciences (Zhejiang Cancer Hospital) Institute of Basic Medicine and Cancer (IBMC), Chinese Academy of Sciences, Hangzhou, Zhejiang Province China; 4 Zhongshan School of Medicine, Sun Yat-sen University, Guangzhou, Guangdong Province China; 5 Cancer Center, Zhejiang University, Hangzhou, Zhejiang Province China

**Keywords:** Metastasis, Ovarian cancer

## Abstract

Peritoneal metastasis is a key feature of advanced ovarian cancer, but the critical protein required for ovarian cancer metastasis and progression is yet to be defined. Thus, an unbiased high throughput and in-depth study is warranted to unmask the mechanism. Transcriptomic sequencing of paired primary ovarian tumors and metastases unveiled that MAP4K4, a serine/threonine kinase belongs to the Ste20 family of kinases, was highly expressed in metastatic sites. Increased MAP4K4 expression in metastasis was further validated in other independent patients, with higher MAP4K4 expression associated with poorer survival, higher level of CA125 and more advanced FIGO stage. Down regulation of MAP4K4 inhibited cancer cell adhesion, migration, and invasion. Notably, MAP4K4 was found to stabilize N-cadherin. Further results showed that MAP4K4 mediated phosphorylation of ADAM10 at Ser436 results in suppression of N-cadherin cleavage by ADAM10, leading to N-cadherin stabilization. Pharmacologic inhibition of MAP4K4 abrogated peritoneal metastases. Overall, our data reveal MAP4K4 as a significant promoter in ovarian cancer metastasis. Targeting MAP4K4 may be a potential therapeutic approach for ovarian cancer patients.

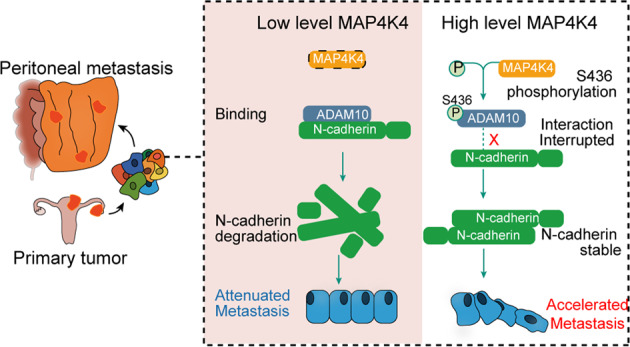

## Introduction

Ovarian cancer is the most frequent cause of cancer death in women and the leading cause of death related to gynecological cancer, accounting for 5% of estimated cancer deaths [1–3]. Due to its asymptomatic nature at early stages, most ovarian cancers were diagnosed at an advanced stage, with distant metastasis in the abdominal cavity [4]. Cancer metastasis is the primary cause of morbidity, and contributes to 95% of cancer-related deaths [5, 6]. Metastatic ovarian cancer is closely related with recurrence and drug resistance, rendering metastasis as the major challenge in the course of ovarian cancer treatment [7]. Thorough understanding of ovarian cancer metastasis is believed to contribute to improve cancer cure rates, while it is still not well elucidated. One important aspect of ovarian cancer metastasis research is the identification of driver molecular. To identify proteins required for ovarian cancer metastasis, we carried out unbiased high-throughput screening by comparing the expression profile of ovarian cancer primary and metastasis tissues. Among the genes identified, Mitogen-Activated Protein Kinase Kinase Kinase Kinase 4 (MAP4K4), also known as HGK (hematopoietic progenitor kinase/germinal center kinase-like kinase) [8, 9], was found to be the most significant kinase associated with metastasis in ovarian cancer.

MAP4K4, a serine/threonine kinase related to the Ste20 family of kinases [8, 9], was reported to promote the migration of ovarian cancer cell SKOV3 [10]. Emerging evidence strongly indicated that MAP4K4 play an important role in cancer and many other biological progresses. *MAP4K4*^−/−^ embryos exhibit the failure of mesodermal and endodermal cells to migrate to their correct location [11]. MAP4K4 was also reported to play a central role in focal adhesion dynamic [12], pancreatic tumorigenesis [13], chemosensitivity in cervical cancer [14], epithelial-mesenchymal transition and metastasis in hepatocellular carcinoma [15] and tumor maintenance in lung adenocarcinoma [16]. However, the detail mechanism and more straight evidence deciphering the promigratory effect of MAP4K4 in ovarian cancer are still lacking.

In this study, we identified MAP4K4 as a critical driver for ovarian cancer metastasis. Further investigation demonstrated that MAP4K4 stabilizes N-cadherin. A disintegrin and metalloprotease 10 (ADAM10) phosphorylation at Ser436 by MAP4K4 consequently reduced ADAM10-mediated down-regulation of N-cadherin. Moreover, in vivo xenograft mouse models, intraperitoneal injection of MAP4K4 inhibitor GNE-495 significantly suppresses the metastasis of ovarian cancer. Our data thus suggest the cancer promoting role of MAP4K4 in ovarian cancer metastasis and its potential as a therapeutic target.

## Materials and methods

### Patients and specimens

Tumor tissues, human omentum and paraffin sections were collected from the Women’s Hospital School of Medicine, Zhejiang University with Institutional Review Board (IRB) approval (approval no. IRB-20200135-R). Informed consent was obtained from all the included patients. All procedure was conducted in accordance with Declaration of Helsinki.

### Cell culture and reagents

Human ovarian cancer cell line A2780 were kindly gifted by the Cancer Research Institute, Zhejiang Cancer hospital (Hangzhou, China). Human ovarian cancer cell lines SKOV3 were purchased from the Culture Collection of the Chinese Academy of Sciences (Shanghai, China). These cells were authenticated by DNA(STR) profiling by iCell Bioscience Inc (Shanghai, China). SKOV3 was cultured with McCoy 5 A, HEK293T cell was cultured with DMEM, IOSE80 AND A2780 was cultured with RMPI 1640 medium. Human peritoneal mesothelial cells were isolated from the tumor-free omentum of patients without malignancy according to the methods described in another study [17]. Primary peritoneal mesothelial cells were cultured on collagen-coated plates with RPMI-1640 media. All media were supplemented with 1% penicillin-streptomycin and 10% FBS and incubated in 37 °C humidified atmosphere with 5% CO2. Cell Proliferation and Toxicity Detection Kit (CCK-8, CK101-01, DIB Data Inventory Biotechnology) were used according to manufacturer’s instruction. ADAM10 inhibitor GI254023X (S8660) was purchased from Selleck. F-actin was visualized with Actin-Tracker Red-Rhodamine (C2207S, Beyotime).

### Plasmids and siRNA and transfaction

Powerfect (SL100569, SignaGen Laboratories) were used in the transfection of siRNA. The details of siRNA sequences are provided in Supplementary Table 1. Plasmids were transfected with Lipo3000 (Thermo Fisher Scientific, USA). The sequences of guide RNAs were gMAP4K4 #1 CAGGACATGATGACCAACTC and gMAP4K4 #2 GGGCGGAGAAATACGTTCAT. Lentivirus packaging was performed in HEK293T cells by transfection of plasmid (lentiCRISPR v2) together with packaging vectors (pMD2.G and psPAX2) using Lipo3000. 48 h after transfection, the medium supernatant containing lentivirus particles was collected and filtered through 0.45 μm filters. Then the viruses together with Polybrene (7 μg/ml, C0351, Beyotime, China) was added were used to infect the target human ovarian cancer cells. After 48 h of infection, the cells were treated with 1 μg/ml puromycin (ST551, Beyotime, China) for 5 days to obtain positive cells. Site mutant plasmid were construct by overlap PCR with primer designed by Takara Primer design tool (https://www.takarabio.com/learning-centers/cloning/primer-design-and-other-tools).

### Cell transwell assay

The cell invasion assay was performed using a 24-well Transwell chamber (Corning, USA). Cells were harvested and suspended in 100 μl serum-free medium were seeded into the upper chamber with an 8 μm pore size insert pre-coated with Matrigel Matrix (356234, Corning). The lower chamber was filled with 600 μl medium containing 10% FBS. After incubation for 18 h, cells were stained with a 0.4% crystal violet solution. The invading cells were imaged randomly using a digital microscopy (Carl Zeiss Jena, Germany). The number of cells were counted in 5 randomly selected fields.

### Wound healing assay

After cells reached 100% confluence, monolayer cells were wounded by scratching the surface straightly as uniformly as possible with a sterilized 200 μl pipette tip. Then the wells were rinsed three times with PBS and replaced with indicated serum-free media. Images were captured by a digital microscopy (Carl Zeiss Jena, Germany) at each indicated time. Experiments were performed in triplicate.

### Protein structure analysis

Z-docking server were used to carry out protein structure analysis [18]. Free energy was calculated with PDBePISA (https://www.ebi.ac.uk/msd-srv/prot_int/pistart.html). The results were visualized in Pymol (The PyMOL Molecular Graphics System, Version 2.0 Schrödinger, LLC) or UCSF ChimeraX (version 1.4 University of California).

### Western blots

The cells were collected and lysed with NP-40 Lysis Buffer (#P0013F, Beyotime, China). Samples of the lysates were separated on 7%–13.5% SDS-PAGE gels and transferred to nitrocellulose filter membranes. Membranes were incubated overnight with primary antibodies, including MAP4K4 (#3485, 1:1000, CST), ADAM10 (#14194, 1:1000, CST), N-cadherin (#13116, 1:1000, CST; 22018-1-AP, 1:1000, Proteintech), vimentin (#5741, 1:1000, CST), pan Phospho-Serine/Threonine Rabbit Polyclonal Antibody (AF5725, 1:1000, Beyotime), HA (#3724, 1:1000, CST), FLAG (#14793, 1:1000, CST), β-actin (AF2811, 1:5000, Beyotime). The next day, membranes were incubated with a secondary horseradish peroxidase-conjugated anti-rabbit or anti-mouse IgG antibody, and then specific bands were visualized by ChemiDoc™ Touch Imaging System (Bio-Rad, USA) using the ECL substrate (P0018FS, Beyotime). β-actin was used as loading control. Experiments were performed in triplicate. Phos-tag™ Acrylamide (304-93521, NARD, Japan) were used in phos-tag gels SDS–PAGE gels.

### Cell adhesion assay

Two methods were employed to examine the adhesion ability of cells. First, 96-well plate was precoated with 10ug/ml fibronectin (HY-P70593, MCE) at 4 °C for 8 h. Then, the plate was blocked with culture medium at 37 °C for 60 min. A total of 20,000 cells per well were then seeded to the plate, followed by incubation at 37 °C for 30 min. Then the supernatant was removed and washed twice with PBS. Subsequently, adhesion cells were fixed with 4% paraformaldehyde at room temperature for 15 min, followed by stained with crystal violet for 10 min. Redundant crystal violet were washed away with mild water flow. Finally, cells were photographed and lysed with 2% SDS at room temperature for 30 min. To quantitatively compare adhesion ability, the absorbance of the lysis at a wavelength of 590 nm were recorded. Second, to mimic the adhesion progression of disseminating ovarian cancer cell to the mesothelial cells in the abdominal cavity, monolayers of primary mesothelial cells were cultured with 96-well plate until complete convergence was reached. Then the supernatant was removed and washed twice with PBS. ZsGreen-labeled SKOV3 cell, with a density of 20,000 cells per well, were seed onto the mesothelial monolayers. Cells were incubated for 30 min followed by three times PBS washes. The protocol was adopted from a published article [19]. Then the fluorescence intensity adhesive ZsGreen expressing cells were quantitatively evaluated with SpectraMax iD5 (Version 2017.12, Molecular Devices, USA).

### In vivo tumor growth in xenograft model

The in vivo ovarian xenograft model was performed using 5-week-old female BALB/c nude mice. Mice were randomly assigned to each group (each group *n* = 5). For each mouse, 1 × 10^6^ SKOV3 cells were injected intraperitoneally. To test the effect of MAP4K4 inhibitor GNE-495, two weeks after SKOV3 cell injection, GNE-495 (3 mg/kg, HY-100343, MCE) were injected for the treatment group daily. The control group were injected with vehicle. The mice were sacrificed 6 weeks after injection, and tumor were counted and collected for further study. Mice were dissected and images were captured under fluorescence stereomicroscope (SMZ18, Research Stereo microscopes, Nikon, Japan). The study was approved by the Ethics Committee of Zhejiang University (Approval Number ZJU20220185).

### Co-immunoprecipitation (Co-IP) and in vitro kinase assay

Co-IP lysis buffer (20 mM Tris-HCl, 150 mM NaCl, 1.5 mM EDTA, 0.5 mM NaVO_4_, 0.5% NP-40, pH = 8.0) with complete protease inhibitor were used for harvest whole cell lysate. Cell lysates were incubated with antibody overnight at 4 °C. The next day, beads were co-incubated with cell lysates and antibody. The beads were washed by washing buffer (20 mM Tris-HCl, 50 mM NaCl, 1.5 mM EDTA, 0.5 mM NaVO_4_, 0.5% NP-40, pH = 8.0), and eluted by elution buffer (Glycine, pH = 2.0) for the Western blot assay.

For in vitro kinase assay, FLAG-tagged MAP4K4 and HA-tagged ADAM10 was overexpressed in HEK293T cells. Proteins were purified by immunoprecipitation with anti-FLAG and anti-HA antibodies. Products were eluted by 1X elution buffer (0.1 M Glycine-HCl, pH 2.5) and 10X neutralizing buffer (0.5 M Tris-HCl, pH7.4, 1.5 M NaCl). Then purified proteins were co-incubated in kinase buffer (25 mM Tris pH 7.5, 2 mM DTT, 10 mM MgCl_2_, 20 uM ATP) at 30 °C for 30 min. The phosphorylated proteins were analyzed in SDS-PAGE.

For analysis of protein phosphorylation by mass spectrometry, SKOV3 cell lysates were immunoprecipitation with anti-ADAM10 antibodies. The obtained sample was subjected to SDS-page and stained with Coomassie brilliant blue. The needed bands were excised and subjected to mass spectrometry (Beijing Qinglian Biotech Co.,Ltd, China.).

### Real-time quantitative PCR

Total RNA was extracted from cells using Trizol reagent (Sangon Biotech, China) according to the manufacturer’s instructions, followed by reverse transcription to cDNA using PrimeScrip™ RT reagent Kit with gDNA Eraser (Takara, Japan). All primers were synthesized by and purchased from the Tsingke Biotechnology Co., Ltd., China. Quantitative real-time PCR was performed using cDNA primers specific for mRNA (7500 Fast Real-Time PCR System, Applied Biosystems, USA). The real-time PCR reactions were performed using Takara’s SYBR Premix Ex Taq™ II (Tli RNaseH Plus) in the Applied Biosystems 7500 Fast Real-Time PCR System (Applied Biosystems, USA). The gene ACTB gene was used as internal control. The details of primer sequences are provided in Supplementary Table 1. Relative transcript abundance of a gene is expressed in ∆Ct values (∆Ct = Ct_reference_ – Ct_target_). Relative changes in transcript levels compared with controls are expressed as ∆∆Ct values (∆∆Ct = ∆Ct_treated_ – ∆Ct_control_). Experiments were performed in triplicate.

### IHC staining

Tumor tissues were fixed by 4% paraformaldehyde and embedded by paraffin. Paraffin embedded tissues were cut into 3-µm sections. Then sections were deparaffinized in dimethylbenzene followed by a graded ethanol series. Sections were then subjected to antigen retrieval by citrate buffer in boiling for 1 h. 5% bovine serum albumin were then used to block the slide for 2 h. Sections were then incubated with 3% hydrogen peroxide at room temperature for 15 min to block endogenous peroxidase activity. Then, the slides were incubated with antibody overnight at 4 °C. The next day, all slides were washed in PBS three times for 5 min each. Secondary antibody was then applied for 2 h at room temperature. DAB solutions, and hematoxylin followed successively. Lastly, all slides were dehydrated and mounted. Images of each slide were captured with BX63 Olympus microscope (Olympus, Japan). Immunostaining intensity was quantified with H-score obtained by multiplying staining intensity (no staining, 0; weak staining, 1; moderate staining, 2 and strong staining, 3) by the percentage (from 0 to 100) of cells under the microscope.

### Public datasets retrieval

Kaplan–Meier Plotter [20] was used to access the association between prognosis and expression level of MAP4K4 and ADAM10 in TCGA and GEO datasets. The following dataset were used in this process: TCGA, GSE19829, GSE26193, GSE27651, GSE26712, GSE30161, GSE9891 and GSE18520. GSE137237 was used to verify the enrichment results. Cancer Cell Line Encyclopedia (CCLE) was used to evaluate the expression profile of kinases among ovarian cancer cell lines, and data were downloaded from Xenabrowser (https://xenabrowser.net/datapages/).

### RNA Seq and enrichment analysis

RNA samples were sequenced on BGISEQ platform (BGI, China). Raw reads were mapped to the reference genome (Homo sapiens, ensemble, GRCh38) using Bowtie2 with the default settings. Numbers of reads assigned to genes were counted by featureCounts [21]. DEGs were retrieved by R package DESeq2 [22]. RNA expression data from RNA-seq was analyzed using GSEA (Version 4.2.3, Broad Institute). Gene signatures were downloaded from the MSIGDB database (software.broadinstitute.org/gsea/msigdb). Hallmark, KEGG and GO gene sets were used.

### Statistics

All statistical analyses were performed using Graphpad Prism (Version 8.0.1, GraphPad Software, LLC) and R (Version 4.1.0, The R Foundation for Statistical Computing). Unless otherwise stated, two-tailed unpaired Student’s *t*-test was used to determine the statistical significance. The Kaplan–Meier method with log-rank testing was used in survival analysis, and the plot was drawn with R package survminer. Scatter plots were created by R package ggpubr. Pearson correlation coefficients with p values were calculated. Heatmaps were drawn with R package pheatmap. Heterogeneity between individual studies was assessed by χ^2^ test and I^2^ test; *p* ≤ 0.05 and/or I^2^ > 50% indicates significant heterogeneity. Summary HRs (RRs) and 95% CI were calculated using a random-effects model when the heterogeneity was significant, and a fixed-effects model was applied otherwise. Two-sided *p* values were calculated, with a *p* value <0.05 considered significant for all tests.

## Results

### High expression of MAP4K4 is associated with peritoneal metastasis and poor prognosis in OV patients

**Fig. 1:**
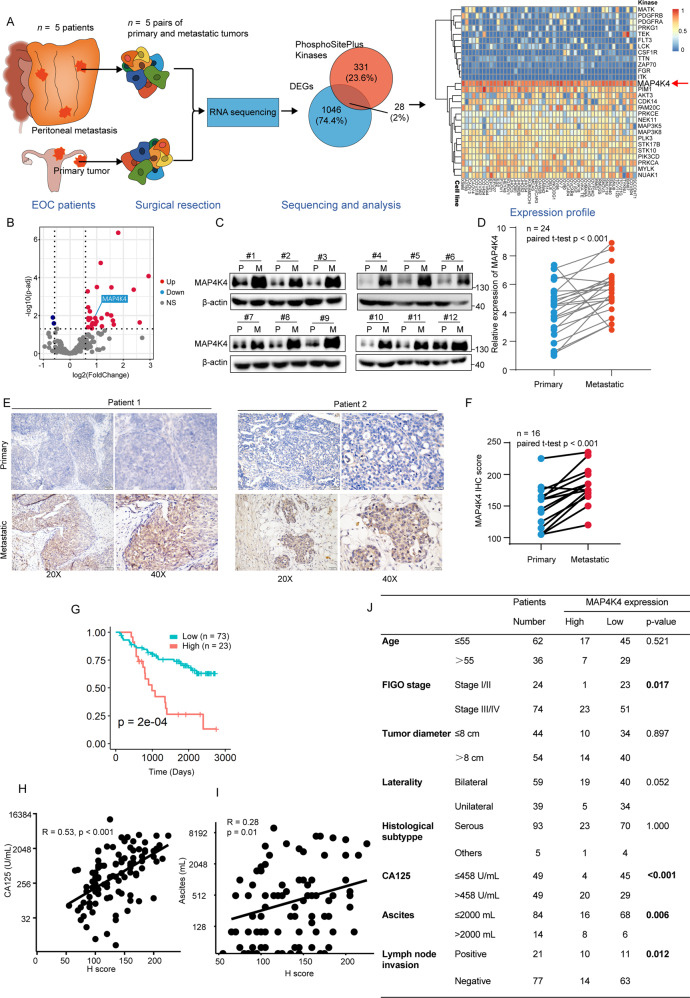
MAP4K4 is highly expressed in peritoneal metastasis and is associated with poor prognosis in OV patients. **A** Diagram for tissue collection and data analysis. **B** Volcano plot shows differentially expressed kinases in peritoneal metastatic tumors as compared to primary tumor based on the diagram in Fig. 1**A**. **C** Western blot analysis of MAP4K4 protein levels in 12 paired ovarian cancer primary and metastatic tissues. **D** The relative mRNA level of MAP4K4 of 24 pairs of ovarian cancer primary and metastatic tissues. **E** Representative images of immunohistochemistry staining for MAP4K4 in ovarian cancer tissues. **F** H-score of IHC staining against MAP4K4 in paired specimen. **G** Kaplan–Meier plot of overall survival for ovarian cancer patients from local cohort stratified by the MAP4K4 expression level. **H** The correlation between MAP4K4 expression and CA125 serum level. **I** The correlation between MAP4K4 expression and ascites volume. **J** Association between clinicopathological characteristics and MAP4K4 expression.

Peritoneal metastases are often the first presentation of ovarian cancer [23]. To figure out the driver kinase for ovarian cancer metastasis, five pairs of ovarian cancer primary tumors and peritoneal metastatic tumors were subjected to transcriptomic sequencing (Fig. 1A). Among 1046 differentially expressed genes, 28 were identified as the potential kinases according to the PhosphoSitePlus (Fig. 1A). The relative expression levels of these 28 genes among ovarian cancer cell lines were displayed in Fig. 1A. Among these proteins, MAP4K4 was universally highly expressed among 50 ovarian cancer cell lines (Fig. 1A). More specifically, MAP4K4 is significantly up-regulated in peritoneal metastasis compared to primary tumor (Fig. 1B). To further validate the differential expression between peritoneal metastases and primary tumors, several independent pairs of peritoneal metastases and primary tumors from local ovarian cancer cohort were subjected for further study. As shown in Fig. 1C, MAP4K4 protein levels were significantly up-regulated in peritoneal metastases. Consistent results were observed at the mRNA level in another 24 independent pairs of tumors (Fig. 1D). We further confirmed that MAP4K4 expression level was higher in metastatic tissues compared with primary tissues (Fig. 1E, F). These findings together suggest that MAP4K4 is highly expressed in ovarian cancer with peritoneal metastases.

Since metastasis is associated with poor prognosis [4, 7, 24], we next aim to study the correlation between MAP4K4 and prognosis of ovarian cancer patients. Kaplan–Meier analysis showed that patients bearing high MAP4K4 level in primary tumors displayed significantly worse prognosis (Fig. 1G). In addition, data from TCGA, GSE19829, GSE26193, GSE27651, GSE26712 and GSE3061 showed that high expression of MAP4K4 was associated with poor prognosis, consistent with our cohort data (Supplementary Fig. 1A–L, Fig. 1G). We also noticed that the expression of MAP4K4 was positively associated with the serum expression of CA125 (Fig. 1H) and ascites volume (Fig. 1I). Detailed information about relationships between clinicopathological factors and MAP4K4 expression were displayed in Fig. 1J. MAP4K4 expression was associated with FIGO stage and lymph node invasion (Fig. 1J), and malignancy (Supplementary Fig. 1M, N).

Collectively, these results imply that MAP4K4 is associated with cancer metastasis and poor prognosis.

### MAP4K4 promotes ovarian cancer cell migration, invasion and adhesion dependent on its kinase activity

To investigate the biological function of MAP4K4 in ovarian cancer progression in vitro, several cell lines were employed. The expression of MAP4K4 in ovarian cancer cell lines was examine by qRT-PCR and immunoblotting. The results showed that MAP4K4 had higher expression in SKOV3 and lower expression in A2780 cells (Supplementary Fig. 2A). Three siRNAs were firstly used to down-regulate MAP4K4 expression inSKOV3 cells (Supplementary Fig. 2B). As siRNA1 and siRNA2 knock down MAP4K4 to a greater extent, they were selected for further study. The results of CCK8 and EdU assay data revealed that *MAP4K4* knock-down did not influence cell proliferation (Supplementary Fig. 2C, D). Colony forming assay revealed the consistent results (Supplementary Fig. 2E).

**Fig. 2:**
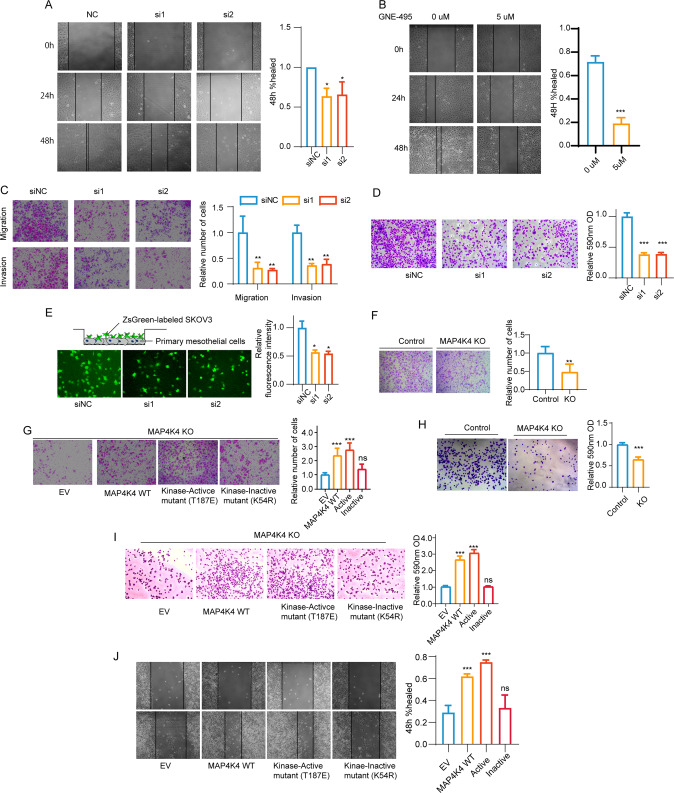
MAP4K4 promotes ovarian cancer cell migration, invasion and adhesion depending on its kinase activity. **A** Wound healing assays of control SKOV3 and *MAP4K4* knock-down SKOV3 cells were performed every 24 h after the cells were scratched. The histograms on the right show the quantitative results of healing percentage. **B** Wound healing assays of vehicle control and 5 uM MAP4K4 inhibitor GEN-495 treated SKOV3 cells were performed. Photos were taken every 24 h after the cells were scratched. The histograms on the right show the quantitative results of healing percentage. **C** Transwell assay for evaluating the migration and invasion of MAP4K4 cells with *MAP4K4* knock down in SKOV3 cells. The histograms on the right show the quantitative results of the number of cells per fields. **D** Representative images of the cell adhesion assay comparing control SKOV3 and *MAP4K4* knock down SKOV3 cells. The histograms on the right show the relative 590 nm OD values. *n* = 5. **E** Representative images of ZsGreen-labeled SKOV3 cells adhering to primary mesothelial cells. The histograms on the right show the relative fluorescence intensity. **F** Transwell assay for evaluating the migration of MAP4K4-KO SKOV3 cells and parent cells. The histograms on the right show the quantitative results of the number of cells per fields. **G** Transwell assay for evaluating the migration of MAP4K4-KO SKOV3 cells with MAP4K4 WT or mutant overexpression. The histograms on the right show the quantitative results of the number of cells per fields. **H** Representative images of the cell adhesion assay in MAP4K4-KO SKOV3 cells and parent cells. The histograms on the right show the relative 590 nm OD values. **I** Representative images of the cell adhesion assay in MAP4K4-KO SKOV3 cells with MAP4K4 WT or mutant over-expression. The histograms on the right show the relative 590 nm OD values. **J** Wound healing assays of A2780 cells with MAP4K4 WT or mutant over-expression. Photos were taken every 24 h after the cells were scratched. The histograms on the right show the quantitative results of healing percentage. Data are presented as the mean ± SD values. Statistical significance was assessed by unpaired *t*-test was performed. *P < 0.05, **P < 0.01, ***P < 0.001, *n* = 3 otherwisely stated.

To assess the effects of MAP4K4 on ovarian cancer cell migration and invasion abilities, wound healing and transwell assays were performed. A delayed wound closure was observed when *MAP4K4* was knocked down (Fig. 2A). The wound healing was also significantly delayed when cells were treated with GNE-495, a specific inhibitor of MAP4K4 (Fig. 2B). Further results showed that the migration and invasion abilities were decreased when *MAP4K4* was knocked down (Fig. 2C).

Ovarian cancer is characterized by its peritoneal dissemination, which is closely related to metastasis and cancer recurrence [4, 25]. To mimic the effect of MAP4K4 on the adhesion behavior of ovarian cancer cells, adhesion assays were performed. The results showed that cell adhesion to laminin coated plate was significantly decreased when *MAP4K4* was knock-down (Fig. 2D). To determine whether MAP4K4 affects the adhesion of ovarian cancer cells to mesothelial cells, ZsGreen-labeled SKOV3 cells were seeded on the monolayer of primary mesothelial cells. As shown in Fig. 2E, *MAP4K4* knock-down reduced the adhesive properties of cancer cells.

To further study the effect of MAP4K4, a SKOV3 cell line with *MAP4K4* knockout (KO) by using CRISPR/Cas9 gene editing was generated. The migration abilities were significantly decreased in *MAP4K4*-KO cells compared with parent cells (Fig. 2F). To understand whether the kinase activity of MAP4K4 is required for such migration abilities, we overexpressed MAP4K4 WT, kinase active mutant or kinase inactive mutant in SKOV3 *MAP4K4*-KO cells. Both MAP4K4 WT and kinase active mutant were able to restore the defective migration abilities of cancer cells, while kinase inactive mutant failed to rescue the defects (Fig. 2G). Consistently, the migration assay revealed that knocking out MAP4K4 inhibited cell adhesion in vitro, and the MAP4K4 kinase activity is also required (Fig. 2H, I). We further showed that overexpression of MAP4K4 WT or kinase active mutant, but not the kinase inactive mutant, was able to rescue the migration defects in A2780 cells (Fig. 2J). To rule out the bias from unequal expression of these WT or mutant constructs, expression level was assessed by WB (Supplementary Fig. 2F, G). No significant differences were observed. The results collectively indicated that the pro-migration effect of MAP4K4 relies on its kinase activity.

Taken together, these findings demonstrated that MAP4K4 promotes ovarian cancer cell migration, invasion and adhesion, which is dependent on its kinase activity.

### MAP4K4 activates epithelial-mesenchymal transition and stabilizes N-cadherin

**Fig. 3:**
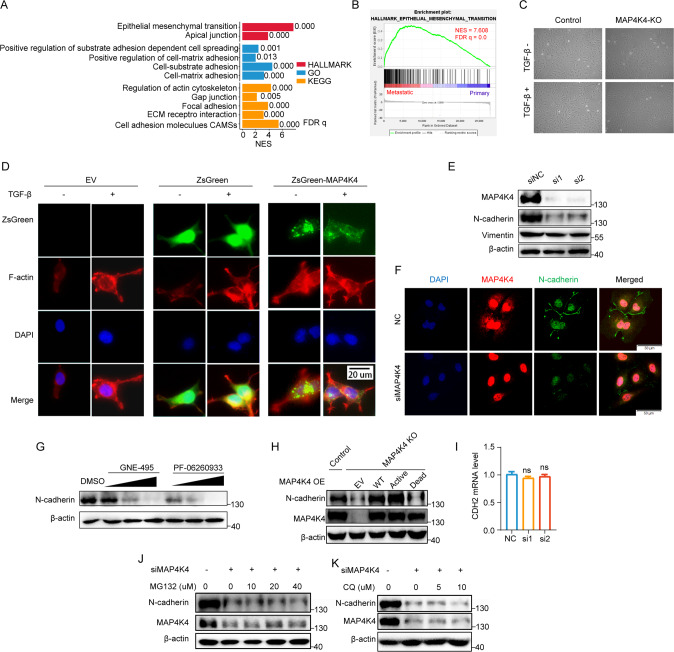
MAP4K4 activates EMT and stabilizes N-cadherin. **A** GSEA results of Metastatic versus Primary tissues. **B** GSEA enrichment plot of hallmark epithelial mesenchymal transition. **C** Morphological change in MAP4K4-KO cell with or without TGFβ(2 ng/mL) treatment for 24 h. **D** MAP4K4 over-expression mimics TGFβ-induced actin organization. Cells were stained for F-actin. TGFβ were applied as 2 ng/mL for 24 h. **E** Western blot analysis of mesenchymal markers in SKOV3 cells when MAP4K4 were knocked down. **F** Immunofluorescence assay of the expression level of N-cadherin in SKOV3 cells with MAP4K4 knockdown by siRNA. **G** Western blot analysis of N-cadherin in SKOV3 cell treated with GNE-495 (2.5, 5, 10 uM for 48 h), or PF-06260933 (5, 10, 20 uM for 48 h). **H** Western blot analysis of N-cadherin in *MAP4K4-*KO SKOV3 cells when MAP4K4 wild-type, kinase active mutant or kinase inactive mutant were over-expressed. **I** The mRNA level of CDH2 (encoding N-cadherin) in the indicated conditions. **J** Western blot analysis of N-cadherin expression. SKOV3 cells were subjected to MAP4K4 siRNA combined with MG132 as indicated concentrations for 6 h. **K** Western blot analysis of N-cadherin expression. SKOV3 cells were subjected to MAP4K4 siRNA combined with CQ as indicated concentrations for 6 h. *P < 0.05, **P < 0.01, ***P < 0.001, *n* = 3 otherwisely stated.

To extract the exact metastasis-related biological processes from genome-wide RNA expression profile, Gene Set Enrichment Analysis (GSEA) was carried out based on the entire network of genes sorted by the log2FC, which was derived from the analysis comparing the expression levels of metastatic tumors with primary tumors. GSEA of RNA sequencing data showed enrichment of signatures covering topics from epithelial mesenchymal transition, cell-substrate adhesion, focal adhesion to cell adhesion molecules CAMs (Fig. 3A, B). These signatures were consistent with enhanced tumor progression in patients. Among them, an epithelial-mesenchymal transition (EMT) signature was the most significantly enriched in the metastatic tissues with an enrichment score of 7.61 (Fig. 3B). Consistent results were obtained in another study (GSE137237) which showed that the epithelial-mesenchymal transition (EMT) signature was also the most significantly enriched in the metastatic tissues with an enrichment score of 6.055 (Supplementary Fig. 2H, I).

TGF-β (transforming growth factor-β) functions as a tumor promotor by inducing epithelial-mesenchymal transition [26, 27]. To verify the up-regulation of EMT process, TGF-β was used to treat SKOV3 cell as positive control. TGF-β treatment induced an evident morphological transition from epithelial phenotype to mesenchymal-like phenotype in SKOV3 cells (Fig. 3C). Moreover, the EMT phenotype could be induced by overexpression of MAP4K4, evidenced by more stress fiber formation and more cellular pseudopodia on cell surface, similar to the effects of TGF-β treatment (Fig. 3D). As Vimentin and N-cadherin are the mesenchymal marker of EMT, we further examined the influence of MAP4K4 on their expression. N-cadherin dramatically decreased upon *MAP4K4* knockdown or knockout (Fig. 3E, F). Pharmacological inhibition of MAP4K4 led to decrease of N-cadherin, which is consistent with the effect of *MAP4K4* knock-down (Fig. 3G). Restoration of MAP4K4 in *MAP4K4*-KO cells rescues the reduced N-cadherin expression level (Fig. 3H). Moreover, such reduction is not due to down-regulated transcription as there was no significant changes on *CHD2* mRNA level (encoding N-cadherin) (Fig. 3I). There are two major pathways involved in regulating protein stability, including the ubiquitin-proteasome system and autophagy-lysosomal system [28]. To understand whether they were involved in regulation of N-cadherin level in *MAP4K4*-KO cells, MG132 (proteasome inhibitor) and chloroquine (CQ, lysosome inhibitor) were utilized for subsequent rescue experiments. However, neither MG132 nor CQ was able to restore the N-cadherin level in cells with *MAP4K4* knocked down (Fig. 3J, K). Collectively, these results imply that MAP4K4 activate EMT and stabilize N-cadherin.

### MAP4K4 stabilizes N-cadherin via an ADAM10 dependent manner

**Fig. 4:**
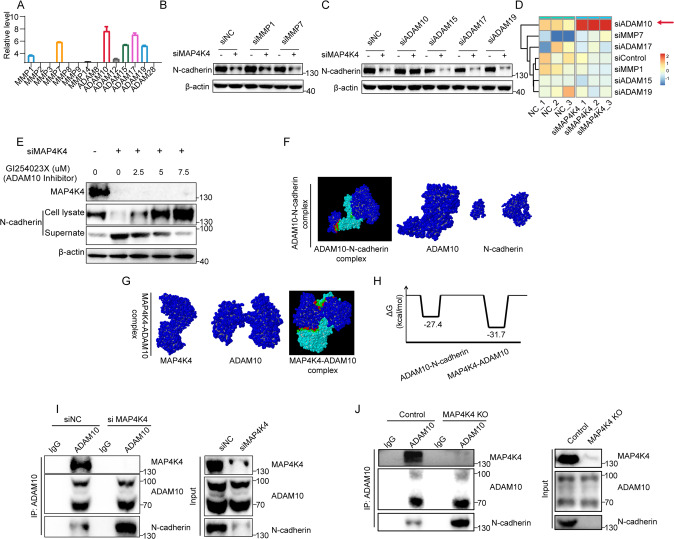
MAP4K4 stabilizes N-cadherin via an ADAM10 dependent manner. **A** Relative mRNA expression of genes encoding metalloproteinases (MMPs and ADAMs) in SKOV3 cells. mRNA expression was normalized to the level of β-actin. **B**, **C** Representative WB results of N-cadherin when MAP4K4 and other MMPs or ADAMs were knocked down. **D** Heatmap and cluster dendrogram of relative level of N-cadherin. The signal intensity in WB results of N-cadherin in triple duplication assays were quantified and normalized. **E** Western blot analysis of the effect of ADAM10 inhibitor GI254023X to N-cadheirn in *MAP4K4* knock-down SKOV3 cells. GI254023X was used with the indicated concentration for 12 h. **F** Molecular modelling to ADAM10/N-cadherin interaction. **G** Molecular modelling to ADAM10/MAP4K4 interaction. **H** The free energy change in indicated complexes. **I** Control cell and siMAP4K4 cells were lysated and immunoprecipitated by anti-ADAM10 antibody. Indicated proteins were checked. **J** Control cells and *MAP4K4*-KO cells were lysated and immunoprecipitated by anti-ADAM10 antibody. Indicated proteins were checked.

Having established the regulatory role of MAP4K4 in N-cadherin stability, we next aim to understand the underlying mechanism. Previous studies reported that metalloproteinases, including matrix metalloproteinases (MMPs) and ADAMs, mediate N-cadherin shedding [29–38]. In order to investigate whether N-cadherin expression level was regulated by metalloproteinases in SKOV3 cell, we examined the expression level of various MMPs and ADAMs in SKOV3 cells. Among them, MMP1, MMP7, ADAM10, ADAM15, ADAM17 and ADAM19 were highly expressed (Fig. 4A). We further investigated the effects of these proteins on the N-cadherin stability and found out that knockdown of *ADAM10*, but not other ADAMs or MMPs, markedly blocked the reduction of N-cadherin upon *MAP4K4* knock down (Fig. 4B, C). ADAM10 is a transmembrane metalloprotease that sheds a range of cell surface proteins including Notch, Eph and N-cadherin [39]. To confirm these results, MMPs/ADAMs screening was carried out by siRNA knock down in triple duplicates. Then, signal intensity was quantified and displayed as heatmap. As shown in the Fig. 4D, *ADAM10* knockdown significantly rescued N-cadherin from *MAP4K4* knock down. The effect of ADAM10 on ovarian cancer prognosis (Supplementary Fig. 3A, B) supported the role of ADAM10 in ovarian cancer.

To further investigate whether ADAM10 is responsible for N-cadherin down-regulation, GI254023X were used to inhibit the activity of ADAM10. Pharmacological inhibition of ADAM10 rescued the reduced N-cadherin level in *MAP4K4* KD cells in a dose-dependent manner (Fig. 4E). To further study the details of the interaction between MAP4K4 and ADAM10, structure modelling was applied. Molecular simulations suggest that ADAM10 and N-cadherin binding cleaves N-cadherin apart (Fig. 4F), which is consistent with previous reports [35, 39]. MAP4K4 not only binds ADAM10 but also leads to a greater decrease in free energy (Fig. 4G, H). We asked whether MAP4K4 stabilizes N-cadherin via disturbing the interaction between ADAM10 and N-cadherin. To test this hypothesis, immunoprecipitation was carried out. The results indicated that the binding of ADAM10 to N-cadherin was enhanced upon *MAP4K4* knock down or knockout (Fig. 4I, J).

The above findings suggest that MAP4K4 may stabilize N-cadherin via an ADAM10 dependent mechanism.

### MAP4K4 binds and phosphorylates ADAM10

**Fig. 5:**
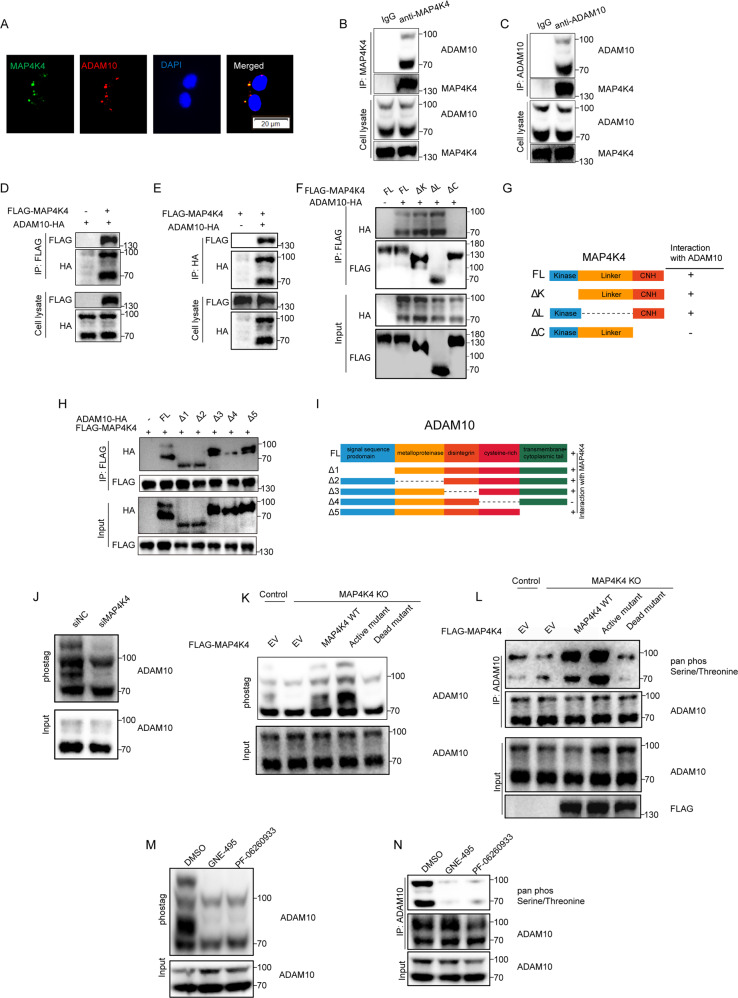
MAP4K4 binds and phosphorylates ADAM10. **A** Immunofluorescence analysis of the localization of MAP4K4 and ADAM10 in SKOV3 cells. **B** Endogenous immunoprecipitation in SKOV3 cell with anti-MAP4K4 antibody. **C** Endogenous immunoprecipitation in SKOV3 cell with anti-ADAM10 antibody. **D** Flag-MAP4K4 and ADAM10-HA were expressed in HEK293T cells, and immunoprecipitated by anti-Flag antibody. **E** Flag-MAP4K4 and ADAM10-HA were expressed in HEK293T cells, and immunoprecipitated by anti-HA antibody. **F**, **G** MAP4K4 binds to the ADAM10 by CNH domain. The diagram on the right illustrate the interaction between MAP4K4 deletion mutants and ADAM10. **H**, **I** ADAM10 binds to the MAP4K4 by cysteine-rich domain. The diagram on the right illustrate the interaction between ADAM10 deletion mutants and MAP4K4. **J** Western blot with phos-tag gel to dissolve phosphorylated ADAM10 after *MAP4K4* knock down. **K** SKOV3 cells were transfected with indicated expression vectors. The cell extracts were subjected to phos-tag gel to dissolve phosphorylated ADAM10. **L** SKOV3 cells were transfected with indicated expression vectors. The cell extracts were subjected to immunoprecipitate HA-tagged ADAM10 and immunoblotted with anti-pan Phospho-Serine/Threonine antibody. The whole cell lysates were blotted with indicated antibodies. **M** Western blot with phos-tag gel to dissolve phosphorylated ADAM10 after treatment with GNE-495 (5 uM for 48 h), or PF-06260933 (10 uM for 48 h). **N** SKOV3 cells were treated with GNE-495 (5 uM for 48 h) or PF-06260933 (10 uM for 48 h). The cell extracts were subjected to immunoprecipitate HA-tagged ADAM10 and immunoblotted with anti-pan Phospho-Serine/Threonine antibody. The whole cell lysates were blotted with indicated antibodies.

We subsequently studied the interaction between MAP4K4 and ADAM10. Endogenous MAP4K4 mutually interacts with ADAM10 (Fig. 5A–C). Consistently, we also overexpressed both MAP4K4 and ADAM10 and confirm their mutual interactions (Fig. 5D, E).

To understand how MAP4K4 interacts with ADAM10, we next performed a mapping assay by generating a series of domain-deletion mutants of both MAP4K4 and ADAM10. The results indicate that MAP4K4 interacts with ADAM10 through its CNH domain, as the ADAM10-HA binding to MAP4K4 CNH domain deletion mutant decreased significantly (Fig. 5F, G). As for ADAM10, the cysteine-rich domain is essential for its binding to MAP4K4 (Fig. 5H, I).

As MAP4K4 belongs to Ste20 family of kinases, we reasoned that ADAM10 may be substrate of MAP4K4 for phosphorylation. To verify this notion, phos-tag gels were used to resolve the phosphor-ADAM10 proteins. When MAP4K4 was depleted, ADAM10 phosphorylation were reduced (Fig. 5J, K). Notably, overexpression of wild-type or the active form of MAP4K4, but not the kinase dead MAP4K4, was able to rescue the reduced p-ADAM10 level in *MAP4K4* KO cells (Fig. 5K). Alternatively, we performed an immunoprecipitation assay with anti-ADAM10 antibody detected the phosphorylation level of ADAM10 with an anti-pan Phospho-Serine/Threonine antibody. Consistent results were observed (Fig. 5L). The ADAM10 phosphorylation was decreased when MAP4K4 was inhibited (Fig. 5M, N).

Taken together, these results demonstrated that ADAM10 interacted with MAP4K4 and was phosphorylated.

### The activity of ADAM10 is suppressed by MAP4K4 mediated phosphorylation at S436

**Fig. 6:**
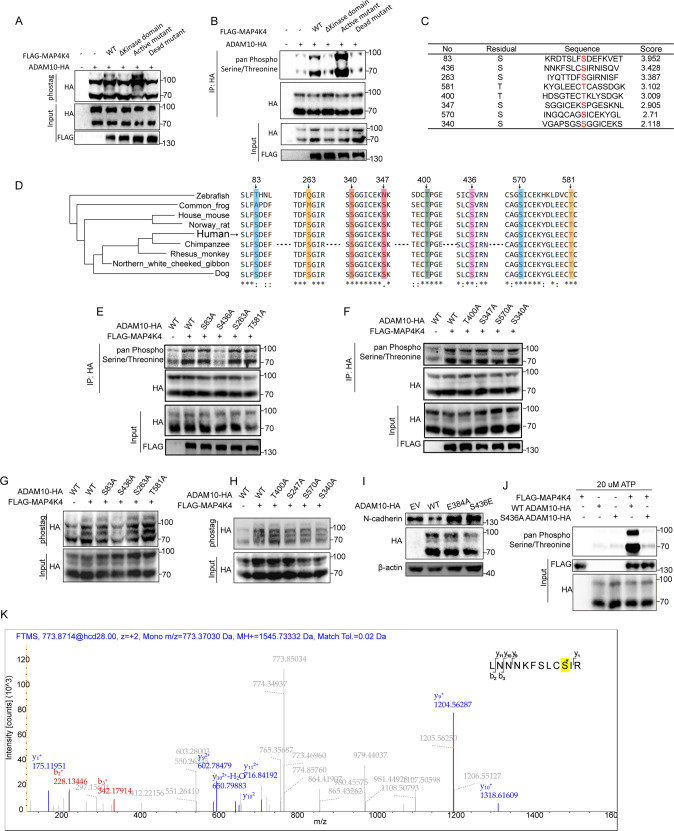
MAP4K4 phosphorylates ADAM10 at S436 and suppresses the activity of ADAM10. **A** HEK293T cells were transfected with indicated expression vectors. The whole cell lysates were loaded to phos-tag gels to resolve the phosphor-ADAM10 proteins, and then immunoblotted with anti-HA antibody. **B** HEK293T cells were transfected with indicated expression vectors. The cell extracts were subjected to immunoprecipitate HA-tagged ADAM10 and immunoblotted with anti-pan Phospho-Serine/Threonine antibody. The whole cell lysates were blotted with indicated antibodies. **C** The potential ADAM10 phosphorylation sites and the corresponding score. **D** Sequence conservation analysis of the relevant amino acids of ADAM10. **E**, **F** HEK-293 cells were transfected with indicated expression vectors. The whole cell lysates were loaded to phos-tag gels to resolve the phosphor-ADAM10 proteins, and then immunoblotted with anti-HA antibody. **G**, **H** HEK293T cells were transfected with indicated expression vectors. The whole cell lysates were loaded to phos-tag gels to resolve the phosphor-ADAM10 proteins, and then immunoblotted with anti-HA antibody. **I** The effect of ADAM10 mutant on N-cadherin expression level. **J** in vitro kinase assay showing S436A mutant could attenuated ADAM10 phosphorylation by MAP4K4 compared with ADAM10 WT. **K** Phosphorylated S436 residues in ADAM10 detected by mass spectrometry analysis of SKOV3 cells.

To verify the phosphorylation of ADAM10 by MAP4K4, Flag tagged MAP4K4 wild type (WT), kinase domain deletion mutant (ΔKinase domain), kinase active mutant, kinase inactive mutant and HA-tagged ADAM10 were over-expressed and their effects on ADAM10 phosphorylation were studied. Phosphorylation of ADAM10 was increased when wild-type MAP4K4 was overexpressed and strikingly enhanced by overexpression of the active form of MAP4K4 (Fig. 6A, B). In contrast, overexpression of MAP4K4 without the kinase domain or the inactive form of MAP4K4 showed marginal effects on ADAM10 phosphorylation (Fig. 6A, B). Therefore, these findings indicated the kinase activity of MAP4K4 is required for ADAM10 phosphorylation.

We next aim to identify the phosphorylation site of ADAM10 mediated by MAP4K4. Software GPS 5.0 [40] was used to predict the potential ADAM10 phosphorylation sites (Fig. 6C, D). Then a series of site-specific ADMA10 mutants were constructed to examine the phosphorylation changes. Among all the mutants, we observed a striking reduction of ADAM10 phosphorylation in the S436A mutant (Fig. 6E, F). Consistent results were observed ADAM10 phosphorylation was detected with phos-tag gels (Fig. 6G, H). Interestingly, S436 fell into the metalloproteinase domain. We thus asked whether the S436 phosphorylation is in control of ADAM10 activity. Then, further assay was carried out to study the effect of S436 phosphorylation on ADAM10’s activity by examining N-cadherin stability. To this end, a catalytically inactive ADAM10 E384A mutant [39] was constructed as a negative control, and ADAM10 S436E mutant was constructed to mimic the phosphorylation of S436. A significant attenuation in N-cadherin degradation was observed in S436E mutant and the E384A mutant, suggesting S436 phosphorylation of ADAM10 may control its activity (Fig. 6I). To determine whether MAP4K4 directly phosphorylates ADAM10, in vitro kinase assay was carried out where purified ADAM10 WT or ADAM10 S436A mutant was incubated with MAP4K4. The results indicated that ADAM10 WT could be phosphorylated, while ADAM10 S436A could not (Fig. 6J). To test whether ADAM10 S436A affect its ability to bind to MAP4K4 and subsequent decrease in phosphorylation, we carried out immunoprecipitation with anti-Flag antibodies. ADAM10 binding to Flag-tagged MAP4K4 was examined by WB. As shown in Supplementary Fig. 3C, no significant decrease of ADAM10 band was observed. Furthermore, mass spectrometry verified S436 as the phosphorylation site of ADAM10 (Fig. 6K).

Collectively, these data demonstrated that MAP4K4 phosphorylates ADAM10 at S436, which depresses the activity of ADAM10.

### MAP4K4 promotes ovarian cancer metastasis in nude mouse xenograft models

**Fig. 7:**
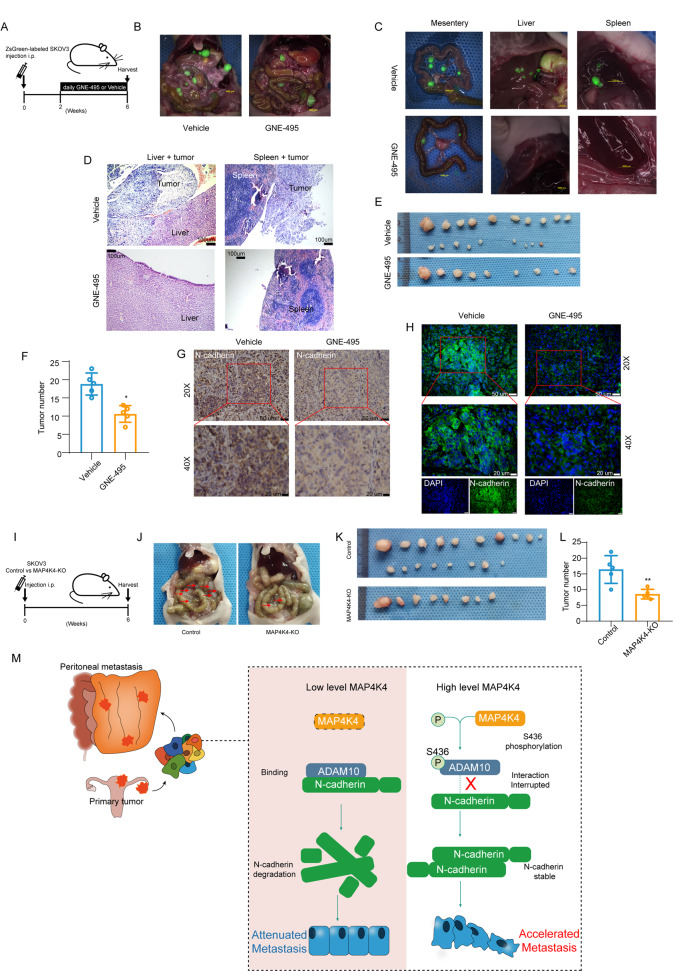
MAP4K4 promotes ovarian cancer metastasis in vivo. **A** Schematic representation of the protocol. **B**, **C** Representative pictures of metastasis in nude mouse xenograft models. **D** HE staining of tumors on the surfaces of liver and spleen. **E** The metastatic tumors were separated, and photos were taken. **F** Quantitative analysis of the number of metastatic tumors in mice treated with Vehicle or GNE-495. **G** Representative images of IHC analyses of tumor specimens from xenograft model using anti-N-cadherin antibody. **H** Representative images of immunofluorescence of tumor specimens from xenograft model using anti-N-cadherin antibody. **I** Schematic representation of the protocol. **J** Representative pictures of metastasis in nude mouse xenograft models. **K** Representative pictures of tumors. **L** Quantitative analysis of the number of metastatic tumors in mice. **M** Schematic representation of ADAM10 S436 phosphorylation by MAP4K4. Phosphorylated ADAM10 contribute N-cadherin stability and therefore promotes ovarian cancer metastasis. Data are presented as the mean ± SD values. Statistical significance was assessed by unpaired *t*-test. *P < 0.05, **P < 0.01, ***P < 0.001, *n* = 5.

To validate the biological function of MAP4K4 in ovarian cancer metastasis in vivo, we performed xenograft models in nude mice. First, MAP4K4 inhibitor GNE-495 was employed to inhibit the activity of MAP4K4. Briefly, two weeks after the injection of SKOV3 cell into abdominal cavity of nude mice, mice injected with GNE-495 (3 mg/kg) or vehicle by intraperitoneal injection daily. After 28 days, mice were sacrificed, with metastasis number determined and tumor tissues were harvested (Fig. 7A).

Peritoneal dissemination of ovarian cells in mice without GNE-495 treatment was much more evident than that in mice with GNE-495 treatment (Fig. 7B), especially in mesentery, spleen and liver (Fig. 7C). HE staining showed that tumor cells invaded into the liver tissue (Fig. 7D), and a mass of tumor cells adhered to the spleen surface (Fig. 7D). As shown in Fig. 7E, GNE-495 treatment reduced the number of peritoneal metastases significantly compared to control (Fig. 7F). In addition, quantitative analysis of the tumor nodes numbers indicated that the control group formed more metastases (Fig. 7F). Immunofluorescence assay and immunohistochemistry staining of N-cadherin indicated that N-cadherin was decreased when treated with GNE-495 (Fig. 7G, H). In order to rule out the nonspecific effect of GNE-495, *MAP4K4*-KO SKOV3 cells and control cells were injected intra-peritoneally into nude mice (Fig. 7I). As shown in Fig. 7J–L, *MAP4K4* knock out significantly reduced the number of peritoneal metastases compared to control. Taken together, these results showed that MAP4K4 promoted ovarian cancer metastasis forming.

## Discussion

Tumor metastasis, the process of tumor cells arising from a primary organ to colonize distant sites, is a major cause to cancer patients’ death [41]. When it comes to ovarian cancer, metastasis remains a clinical challenge for the current ovarian cancer treatment [4, 7]. As a large sac, the peritoneum is a largest and complex serous membrane providing the virtual space filled with a small amount of serous fluid, which is susceptible to ovarian cancer metastasis [24]. Peritoneal metastasis is the most common form of metastasis in advanced ovarian cancer. However, the molecular mechanism driving the metastasis is not fully elucidated. In this study, our transcriptomic sequencing data demonstrated the distinct gene expression profile of metastatic tumors compared with the primary tumors. We identified MAP4K4 as a most overexpressed kinase during peritoneal metastasis, rendering it an important driver for metastasis formation in ovarian cancer (Fig. 1A). We further examined the functional role of MAP4K4 in metastatic progression and demonstrated that MAP4K4 stabilized N-cadherin by suppression of ADAM10 to promote cell adhesion, migration and invasion. Moreover, in vivo xenograft models indicated the therapeutic value of inhibiting MAP4K4 in reducing ovarian cancer peritoneal metastasis. The present study, therefore, proposed a novel mechanism in ovarian cancer metastasis, providing theoretical basis for the possible clinical treatment.

Our results showed that MAP4K4 promoted ovarian cancer cell adhesion, migration and invasion. Consistently, whole-body knockout of *MAP4K4* results in mice lethality between embryonic day E9.5 and E10.5 because of the failure of mesodermal and endodermal to migrate to their correct location [11]. MAP4K4 was also identified as an enhancer of endothelial cell membrane retraction and a regulator of pathologic angiogenesis [12]. The role of MAP4K4 in cell migration was supported by several studies as well. Jiping Yue and colleagues reported that MAP4K4 acts as a microtubule-dependent disassembly factor to promote focal adhesion dynamics [42]. Another study employed large-scale CRISPR-Cas9 loss of function screen and identified MAP4K4 as a strong regulator of glioblastoma invasion [43]. Absence of MAP4K4 leads to the cell transition to a non-invasive state, which is consistent with our study. In prostate cancer cells, MAP4K4 promoted F-actin organization [44]. Besides, the important multiple roles of MAP4K4 in immunity [45, 46], neuropathology [47, 48], metabolic [49], inflammation [50], and cardiovascular disease [51] had been widely recognized. More and more information regarding MAP4K4’s role in cancers had been reported. Significant role of MAP4K4 in cancer progression has been reported seen in cervical cancer [14], pancreatic tumorigenesis [13] and lung adenocarcinoma maintenance [16]. These data indicated that MAP4K4 might act as a novel cancer promoting kinase. Through IHC staining, MAP4K4 intensity was closely associated with poorer prognosis, higher level of CA125, higher level of ascites volume and more advanced FIGO stage (Fig. 1G–J). In the present study, we demonstrate that MAP4K4 plays a pro-metastatic role in ovarian progression (Fig. 2). MAP4K4 promotes ovarian cancer cell migration, invasion and adhesion depending on its kinase activity (Fig. 2F, I, J). During this process, MAP4K4 stabilizes N-cadherin (Fig. 3E, F). In this study, we also evaluated the clinical relevance of MAP4K4 levels with ovarian cancer progression in patient samples. In summary, these data demonstrate a critical functional role of MAP4K4 in ovarian cancer metastasis.

EMT is known to be a crucial biological process to initiate the progression of tumor metastasis cascade. The cadherin switch from epithelial E-cadherin to mesenchymal N-cadherin is a major hallmark of EMT and its mesenchymal markers N-cadherin and Vimentin are frequently being discussed [52–54]. Vimentin and N-cadherin were reported to increase allocation of actin into stress fiber formation and enhance the ability of cancer cells to migrate around [55–57]. However, a genetic fate-mapping system used in studying primary tumor metastasis showed that primary tumor cells activated vimentin and N-cadherin in situ, but tumor cells that have ever expressed N-cadherin contributes to the majority of metastases cells [58], suggesting the important role of N-cadherin in cancer metastasis. In our study, MAP4K4 was identified to stabilize N-cadherin in a posttranslational modification manner (Fig. 3J, K). Previous study has mainly focused on the transcription activation of N-cadherin by EMT activating transcription factors (EMT-TFs) including SNAIL (also SNAI1) and SLUG (also SNAI2), the basic helix–loop–helix factors TWIST1 (TWIST) and TWIST2 and the zinc finger E-Box binding homeobox factors ZEB1 and ZEB2 [59]. In this study, we highlight a novel mechanism in the regulation of N-cadherin.

Recent studies have uncovered an important role of the ADAM10 in a variety of pathophysiological conditions. ADAM10 is comprised of an N-terminal signal sequence and an adjacent prodomain, followed by metalloprotease, cysteine-rich, disintegrin, and cysteine-rich domains domains [39]. With the intact prodomain, the full-length ADAM precursors are catalytically inactive [39]. Dysregulated ADAM10 activity is implicated in a wide range of biologic processes, including immunity [60], lung fibrosis [61], Cell Adhesion [35, 62] and Neurite outgrowth [63]. Particularly, the role of ADAM10 in cancer is widely discussed. In Glioblastoma, ADAM10 promote tumor progression by several proposed mechanisms, including Neuroligin-3 release [64, 65], cleavage of N-cadherin [32], and depression of NK cell activation [66]. ADAM10 contributes to prostate cancer metastasis via cleaving ephrin-A5 [67]. The active ADAM10 form marks cancer stem-like cells in breast cancer [68]. Interesting, considering the role of ADAM10 in N-cadherin cleavage [35] and the promigraion effector of N-cadherin in cancers [69–71], we proposed the hypothesis that ADAM10/N-cadherin might play a role in MAP4K4 mediated N-cadherin stability. The unbiased screen of MMPs and ADAMs in the current study identified ADAM10 as a special substrate involving in MAP4K4 mediated N-cadherin stability (Fig. 4B–D). ADAM10 inhibition rescued the expression level of N-cadherin brought by *MAP4K4* knockdown (Fig. 4B). MAP4K4 mediated ADAM10 phosphorylation at S436 depressed the activity of ADAM10 and hence suppressed N-cadherin shedding (Fig. 6I). Our work demonstrates the effect of post-translation modification in the control of ADAM10 activity. So far, most studies had mainly focused on the effects of prodomain cleavage on ADAM10 activity. In this study, we highlighted the significance of post-translation modification in ADAM10 activity.

The development of druggable targets against tumor metastasis is the goal of basic theoretical research. Based on the pro-metastasis role of MAP4K4 in ovarian cancer, we speculated that MAP4K4 could be a therapeutic target. Kinome scan showed that MAP4K4 activity was reduced by 27% with 10 uM dabrafenib [72]. MAP4K4-specific small-molecule inhibitors have been currently available. Although they have not been tested by clinical trials, these small-molecule inhibitors show promising outcomes in animal models. MAP4K4 inhibitor GNE-495 reduced retinal neovascularization, vascular regrowth, and haemorrhage [12, 73]. Another inhibitor PF-06260933 improved atherosclerotic plaque development in mice fed with a custom diet containing high cholesterol content [74]. PF-06260933 also provided 44% reduction of blood glucose in fasting hyperglycemia [75]. Moreover, attentions have been raised recently on down regulation of MAP4K4 as anticancer agents [76]. The antitumor effects of MAP4K4 silencing were also observed manifested by reduced tumor xenograft growth in hepatocellular carcinoma [77]. GNE-495 decreased the tumor burden and extended survival of the KPC mice with pancreatic cancer [13]. Our data demonstrated that GNE-495 intraperitoneal injection inhibited ovarian cancer metastasis in nude mouse ovarian cancer xenograft models (Fig. 7B–F). In summary, MAP4K4 inhibition by potential drug candidate might be particularly beneficial for ovarian cancer patients.

## Conclusion

In summary, transcriptome profiling has identified MAP4K4 as a novel promoter for ovarian cancer metastasis. MAP4K4 enhances cell adhesion, migration and invasion, stabilizes N-cadherin and promotes in vivo metastasis. MAP4K4 controls N-cadherin stabilization by phosphorylates ADAM10 at Ser436, leading to decreased ADAM10 activity. Taken together, the results indicated that that MAP4K4 is a novel and important anti-metastasis therapeutic target.

## Supplementary information

Supplementary Information

## Data Availability

The accession number for the RNA-seq sequencing and processed data reported in this paper is GEO: GSE222982. Estimated raw counts for all samples were also available in the data repository of our team (http://bigzju.com/data_repository/). Other data needed to evaluate the conclusions in the paper are present in the paper and/or the Supplementary Materials. Additional data and code related to this paper may be available upon reasonable request.
