# Supplementary material for: MAP4K4 promotes ovarian cancer metastasis through diminishing ADAM10-dependent N-cadherin cleavage
Source: Oncogene. 2023 Mar 15;42(18):1438–52. doi: 10.1038/s41388-023-02650-5 (PMC10154218; doi:10.1038/s41388-023-02650-5)
Supplement: Supplementary file 1 — Supplementary Information [file 41388_2023_2650_MOESM1_ESM.docx]

MAP4K4 promotes ovarian cancer metastasis through diminishing ADAM10-dependent N-cadherin cleavage

Kelie Chen^1, 2#^, Xiaoyu Yuan^2#^, Shengchao Wang^1#^, Fang Zheng^1, 2#^, Zhiqin Fu^3^, Zhangjin Shen^1^, Xiaodong Cheng^1^, Yuwei Wang^2^, Song Tang^1, 2^, Heng Ni^2^, Fang Wang^2^, Guang Lu^4^, Yihua Wu^1, 2*^, Dajing Xia^1, 2, 5*^, Weiguo Lu^1, 5*^

The authors declare that they have no known competing financial interests or personal relationships that could have appeared to influence the work reported in this paper.

1 Department of Gynecologic Oncology of Women's Hospital, Zhejiang University School of Medicine, Hangzhou, Zhejiang Province, China

2 Department of Toxicology of School of Public Health, Zhejiang University School of Medicine, Hangzhou, Zhejiang Province, China

3 The Cancer Hospital of the University of Chinese Academy of Sciences (Zhejiang Cancer Hospital) Institute of Basic Medicine and Cancer (IBMC), Chinese Academy of Sciences, Hangzhou, Zhejiang Province, China

4 Zhongshan School of Medicine, Sun Yat-sen University, Guangzhou, Guangdong Province, China.

5 Cancer Center, Zhejiang University, Hangzhou, Zhejiang Province, China

# These authors contributed equally to this work.

Co-corresponding authors: Yihua Wu, Dajing Xia, Weiguo Lu

**Correspondence**

Weiguo Lu, Department of Gynecologic Oncology of Women's Hospital, Zhejiang University School of Medicine, #1 Xueshi Road, Hangzhou, P.R. China

Tel: 86-0571-87061501

Fax: 86-0571-87061878

Email: [lbwg@zju.edu.cn](mailto:lbwg@zju.edu.cn)

Dajing Xia, Department of Toxicology of School of Public Health, Zhejiang

University School of Medicine, #866 Yuhangtang Road, Hangzhou, P.R. China

Tel: 86-0571-88208134

Fax: 86-0571-88988103

Email: [dxia@zju.edu.cn](mailto:dxia@zju.edu.cn)

Yihua Wu, Department of Toxicology of School of Public Health, Zhejiang

University School of Medicine, #866 Yuhangtang Road, Hangzhou, P.R. China

Tel: 86-0571-88208134

Fax: 86-0571-88208140

Email: [georgewu@zju.edu.cn](mailto:georgewu@zju.edu.cn)

**Supplementary Figures and Legends**


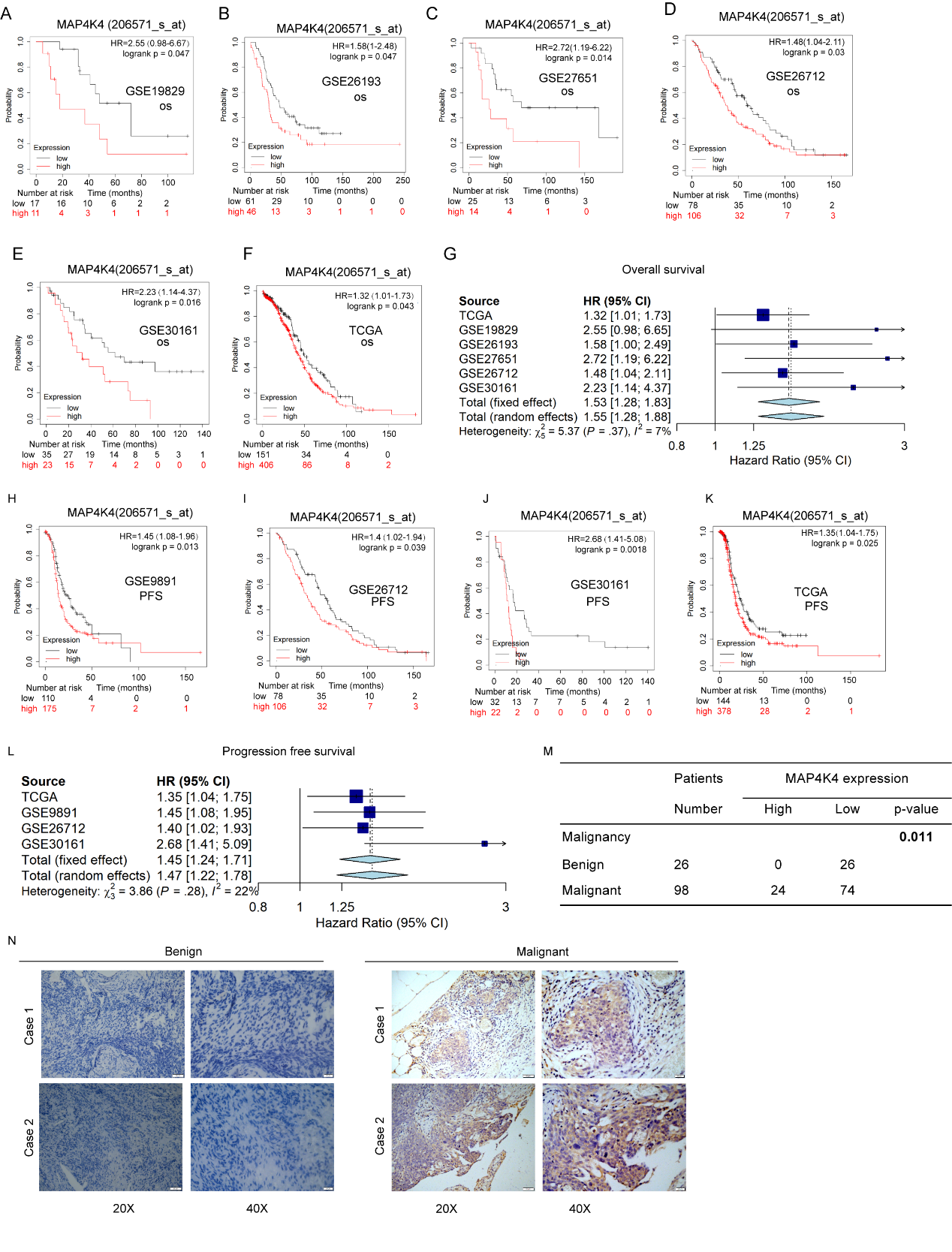


**Supplementary Figure 1** **MAP4K4 is associated with poorer prognosis.** (A-F) Kaplan–Meier curves of overall survival based on data from GSE19829(A), GSE26193(B), GSE27651(C), GSE26712(D), GSE30161(E) and TCGA(F). (G) Forest plot of HRs from SF1 A-F. (H-K) Kaplan–Meier curves of progression free survival based on data from GSE9891(H), GSE26712(I), GSE30161(J) and TCGA(K). (L) Forest plot of HRs from SF1 H-K. (M) Association between malignancy and MAP4K4 expression. (N) Representative images of immunohistochemistry staining for MAP4K4 in ovarian tumors.


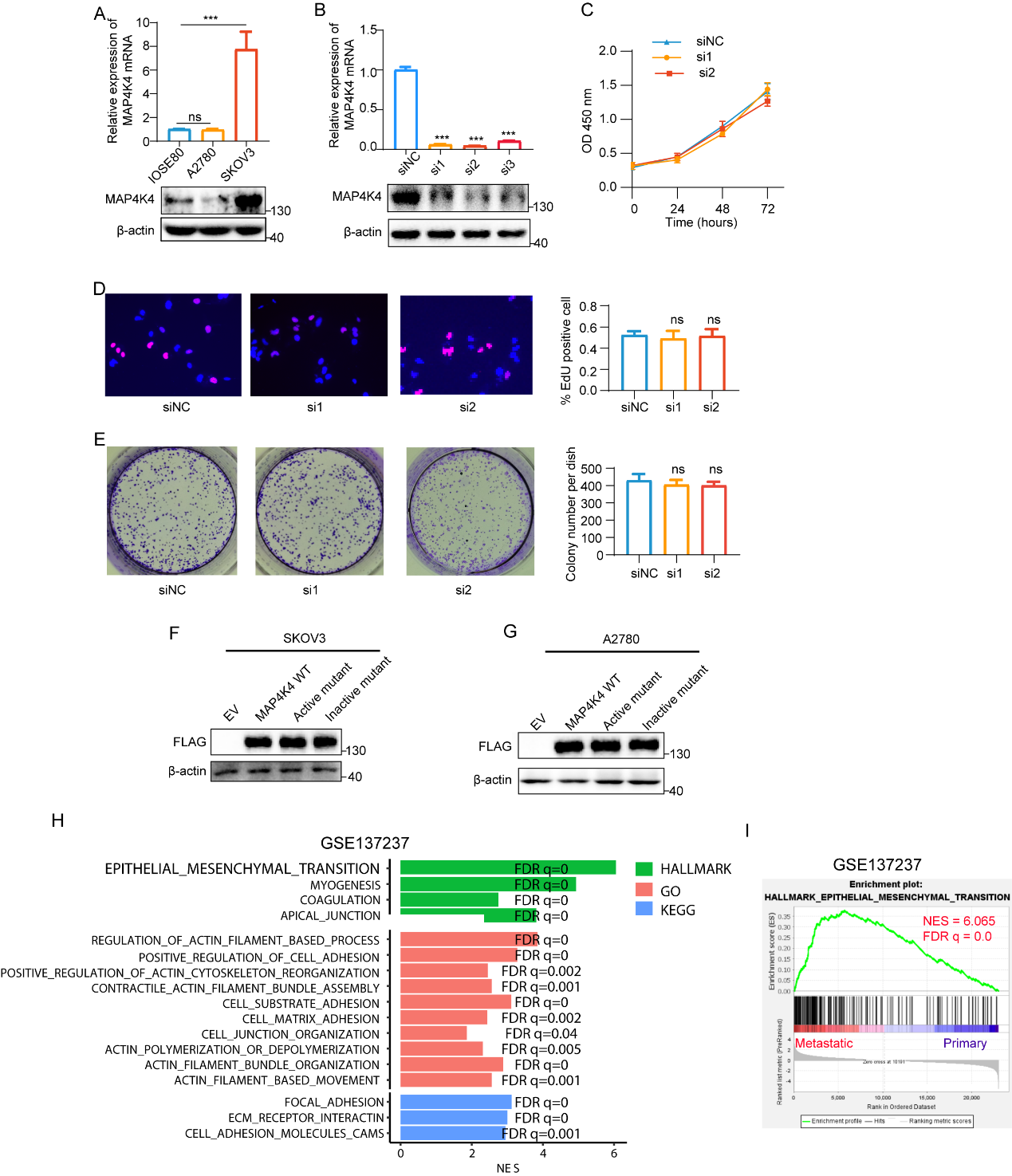


**Supplementary Figure 2 The effect of MAP4K4 in cell proliferation and GSEA results of GSE137237**.

(A) Relative expression of MAP4K4 in different cell lines. (B) Western blot analysis of MAP4K4 in SKOV3 cells by MAP4K4 knock down. (C) EdU assay for evaluating the proliferation abilities of MAP4K4 knockdown in SKOV3 cells. The histograms on the right show the quantitative analysis results of positive cells. (D) Colony formation assays of MAP4K4 knockdown in SKOV3 cells. The histograms on the right show the quantitative analysis results of positive cells. (E) Cell proliferation of the control and MAP4K4 knock down SKOV3 cells was assessed every 24 h for 3 days using the CCK8 assay. (F) The expression level of different constructs in SKOV3 cells. (G). The expression level of different constructs in A2780 cells. (H) GSEA results of Metastatic versus Primary tissues in GSE137237. (I) GSEA enrichment plot of hallmark epithelial mesenchymal transition in GSE137237. *P < 0.05, **P < 0.01, ***P < 0.001, ns: non-significant, n = 3.


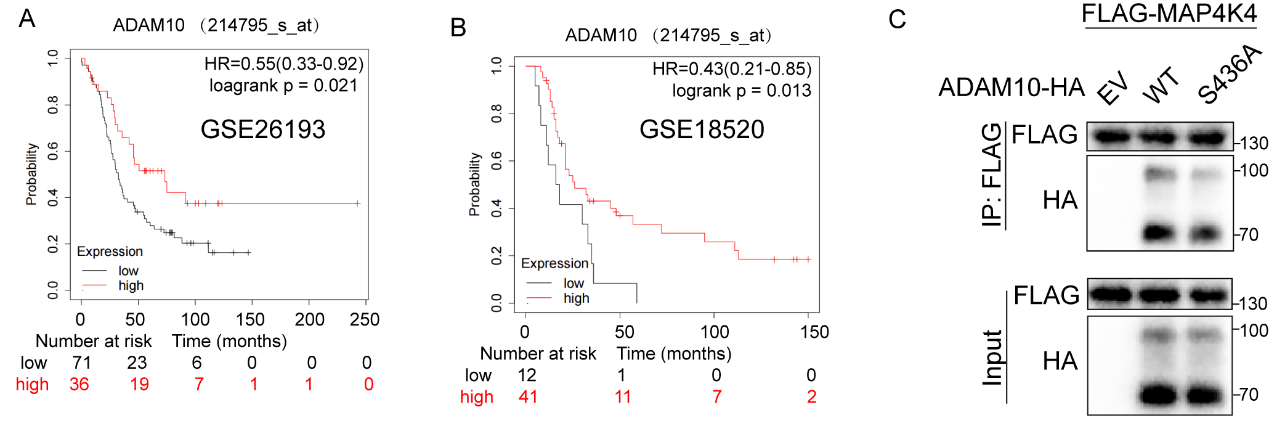


**Supplementary Figure 3 ADAM10 is associated with poorer prognosis.** (A-B) Kaplan–Meier curves of overall survival based on data from GSE26193 (A) and GSE18520 (B). (C) Flag-tagged MAP4K4 and HA-tagged ADAM10 WT/mutant constructs were expressed in HEK293T cells, and immunoprecipitated by anti-Flag antibody.

**Supplementary Table 1**

**siRNA sequences**

| **Gene** | **sense** | **antisense** |
| --- | --- | --- |
| MAP4K4 #1 | GAUGACCAACUCUGGCUUGtt | CAAGCCAGAGUUGGUCAUCtt |
| MAP4K4 #2 | CGCAAUGACAAGGUGUUCUtt | AGAACACCUUGUCAUUGCGtt |
| MAP4K4 #3 | UAUAAGGGUCGACAUGUUAtt | UAACAUGUCGACCCUUAUAtt |
| MMP1 | GCUGCUUACGAAUUUGCCGtt | CGGCAAAUUCGUAAGCAGCtt |
| MMP7 | GGCAUUCAGAAACUAUAUGtt | CAUAUAGUUUCUGAAUGCCtt |
| ADAM10 | GACAUUUCAACCUACGAAUtt | AUUCGUAGGUUGAAAUGUCtt |
| ADAM15 | GCAAAUGCCAUGGACAUGGtt | CCAUGUCCAUGGCAUUUGCtt |
| ADAM17 | GUGCCAGGAGGCGAUUAAUtt | AUUAAUCGCCUCCUGGCACtt |
| ADAM19 | CCACCACACGGAAAUUGGAtt | UCCAAUUUCCGUGUGGUGGtt |

**Primer sequences for PCR**

| **Gene** | **F** | **R** |
| --- | --- | --- |
| MMP1 | GGTCTCTGAGGGTCAAGCAG | AGTTCATGAGCTGCAACACG |
| MMP2 | ACAGCAGGTCTCAGCCTCAT | TGAAGCCAAGCGGTCTAAGT |
| MMP3 | GCAGTTTGCTCAGCCTATCC | GAGTGTCGGAGTCCAGCTTC |
| MMP7 | GAGTGCCAGATGTTGCAGAA | AAATGCAGGGGGATCTCTTT |
| MMP8 | TCTGCAAGGTTATCCCAAGG | CTTGCTGGAAAACTGCATCA |
| MMP9 | TCTGCAAGGTTATCCCAAGG | CTTGCTGGAAAACTGCATCA |
| MMP14 | CAGAGAAGGCACACAAACGA | CACTGGTGAGACAGGCTTGA |
| ADAM8 | ACCCTTCCCAGTTCCTGTCT | CTCCTTGCTTCCTCTGGATG |
| ADAM9 | GTTCCTGTGGAGCAAAGAGC | CCAGCGTCCACCAACTTATT |
| ADAM10 | AGCAACATCTGGGGACAAAC | CCCAGGTTTCAGTTTGCATT |
| ADAM12 | GCCTCTGTCTTCTGGACTGG | TGGAAGGCAAACCAAGATTC |
| ADAM15 | AGCCTCAAAAAGGTGCTTCA | CCCTGGTAGCAGCAGTTCTC |
| ADAM17 | TGAGGGCAGTTAACCAAACC | ATACACCCACACACCCCACT |
| ADAM19 | CTGAAGGCTGTGGGAAGAAG | ACCACAGGACCCACACTCTC |
| ADAM28 | TTCAGTCAGGGGGATCAAAG | TAGTTTGGTGGCAGGTAGGG |
| MAP4K4 | GGAACACACTCAAAGAAGACTGG | GTGCCTATGAACGTATTTCTCCG |
| CDH2 | TCAGGCGTCTGTAGAGGCTT | ATGCACATCCTTCGATAAGACTG |
| ACTB | ATCATGAAGTGTGACGTGGA | CTCAGGAGGAGCAATGATCT |
